# Homicide in Relation to Mental Illness: Stigma Versus Reality

**DOI:** 10.7759/cureus.32924

**Published:** 2022-12-25

**Authors:** Zainab A Almomen, Abdullah H Alqahtani, Lina A Alafghani, Ali F Alfaraj, Ghaida S Alkhalifah, Nawal H Bin Jalalah, Norah A Alsuwailem, Rawan M Hilal

**Affiliations:** 1 Family Medicine, Dammam Medical Complex, Ministry of Health, Dammam, SAU; 2 Psychiatry, Johns Hopkins Aramco Healthcare (JHAH), Dhahran, SAU; 3 General Practice, Ministry of Health, Al-Ahsa, SAU; 4 Internal Medicine, King Abdulaziz University (KAU), Jeddah, SAU; 5 General Practice, Dammam Medical Complex, Ministry of Health, Dammam, SAU; 6 Family Medicine, Ministry of Defense, Dhahran, SAU; 7 Psychiatry, Dammam Medical Complex, Ministry of Health, Dammam, SAU

**Keywords:** weapons, motives, stigma, characteristics, mental illness, homicide

## Abstract

This narrative review examines different aspects of homicide among mentally ill individuals to compare the rates of homicide by offenders with and without mental illness and investigate the stigma of mental illness and its consequences. It also evaluates the motives of mentally ill perpetrators and their characteristics and explores weapons of choice in homicides related to different mental disorders. Studies confirmed higher homicide rates among specific categories of mentally ill individuals who experienced maltreatment, unemployment, abuse in childhood, and substance abuse resulting from stigma and discrimination. The motives were mainly revenge, argument, financial gain, sexuality, sadism, and filicide, with revenge being the top motive. Offenders were found to have close relationships with their victims in most cases. Sharp instruments were the most commonly used weapons. Our review confirms the lack of evidence linking mental illness independently with homicide, both globally and in Arab countries, and highlights the impact of discrimination toward mentally ill individuals. This discrimination and stigma lead to delayed care-seeking and self-destructive behavior, which is linked to higher homicide rates among persons with and without mental illness.

## Introduction and background

Homicide is the most tragic form of violent crime. It refers to the act of taking another human being’s life with the intent to cause death, whether killing is lawful or unlawful. Therefore, homicide is considered a general term that can be classified into two types: criminal and noncriminal acts of murder. Distinguishing between homicide types is essential for applying justice because penalties vary according to the circumstances of the act and the killer’s intentions and behaviors. For example, punishments could be either life imprisonment or capital punishment, which are associated with crimes of clear intent. European legal systems, such as Anglo-American systems, rely on the murderer’s intention as an essential element. However, the emphasis is more on the circumstances of the act and the killer’s conduct. However, Islamic law considers murder a civil infraction, giving the family of a murdered victim the right to choose between taking retribution (Qiṣāṣ) and accepting wergild (Diyah) [1].

Homicide rates in different countries vary but can never be zero. However, it is difficult to determine the prevalence of homicide in a specific area within a particular year. In fact, the recorded criminal cases only represent the “tip of the iceberg”; this concept is known as the “dark figure,” which is a common problem not only in forensic science but in all social sciences [2]. In 2017, more than 400,000 people died of homicide globally. Homicide was one of the leading causes of death in some countries of Latin America, being third in Venezuela, fourth in Honduras, and fifth in Guatemala. Generally, the rate of homicide across most countries is less than 1%, but some countries had significantly higher rates, such as Honduras (9%), Venezuela (8%), Guatemala (7%), and Mexico (6%). In contrast, the rates in Middle Eastern countries were much lower: Kuwait (0.5%), Bahrain (0.63%), Qatar (0.57%), United Arab Emirates (0.44%), and Saudi Arabia (0.32%), with a total homicide rate of 0.9 deaths per 100,000 people in 2017 [3]. In Dammam, a retrospective study conducted at the Forensic Medicine Center between 2006 and 2010 aimed to provide a database associated with injury mortality and generate a system that helps record information concerning homicidal deaths. This study included 125 homicide victims, of whom 104 were men and 21 were women (83.2% versus 16.8%). The average age of most victims (77.6%) was between 21 and 50 years of age, and most had blunt force injuries. During the span of five years, 2009 was associated with the highest rate of homicide, which represented 29.6% of the 125 cases. The total annual homicide rate was 1.49 per 100,000 people [4].

As mentioned previously, homicide is a broad term that can refer to different cases in different circumstances. Our review concentrates on criminal homicides among individuals with mental disorders. According to a retrospective study of homicide in New Zealand from 1970 to 2000, 8.7% of 1498 homicides were committed by mentally abnormal offenders. In this study, murderers were referred to as “mentally abnormal homicide offenders” when the killer was a known case of a serious mental disorder and represented one of the four groups in courts: unsuitable to undergo trial, blameless due to insanity, pronounced guilty of infanticide, or condemned to psychiatric committal [5]. In this narrative review, we aimed to compare the rates of homicide by offenders with and without mental disorders, investigate the stigma of mental disorders and its consequences, evaluate the motives of mentally ill perpetrators and their characteristics, and explore weapons of choice in homicides related to different mental disorders.

## Review

Homicide, stigma, and mental disorder

Mental disorders have been correlated with stigma over decades despite increasing mental health awareness. A national survey which was conducted between 1950 and 1996, then repeated in 2006, showed that stigma towards mental illness continued to be one of the major problems in society despite the increase in knowledge and education about mental disorders [6]. In 1999, the same finding was reported by the Surgeon General, which shows that, over the past 40 years, stigma kept increasing toward mentally ill people regardless of the improvement in public awareness of mental illnesses [7]. Stigma complicates mentally ill persons’ lives by reducing their self-efficacy and self-esteem, leading to a sense of worthlessness. Therefore, mentally ill individuals tend to undermine their efforts to succeed in life, such as obtaining a permanent job. However, it enhances social, personal, and economic aspects of their lives in addition to clinical state improvement [8]. Stigma not only leads to unemployment but also delays presentation and care-seeking [9]. Unfortunately, the stigma and ignorance rates are high in Arab countries [10]. According to WHO, up to 85% of individuals with mental disorders are untreated, especially in low- and middle-income countries [11]. Although the public believes that individuals suffering from severe mental illnesses are criminals, they are likely to be victims of stigma and discrimination [8]. In fact, mentally ill individuals are more likely to harm themselves than harm others, as suicide risk is 20 times greater among mentally ill individuals than among the general population [12]. For instance, in a study from China investigating the prevalence of violence, runaway, and suicide among individuals with schizophrenia, suicide had the lowest rate (4%), followed by runaway (6.5%) and violence (17.25%). Despite the low prevalence of suicide, it is still considerably higher than the general population’s prevalence, which is 0.9% [13]. Mentally ill individuals have symptoms that include impulsivity, disorganized thoughts, and impaired reality testing; hence, their ability to perceive risks is diminished, and they are more vulnerable to physical assaults [14]. In a study conducted in Wales and England involving all individuals who committed homicides between 1990 and 1996, 34% of the perpetrators had a mental disorder. Most participants did not receive mental health services. Furthermore, only 10% of patients had symptoms of mental illness during the offense [15]. Similarly, a Russian study involving 3414 homicide perpetrators, who were evaluated by experienced psychiatrists before their trial, showed that 46.7% of them met at least one of the diagnostic criteria of the International Classification of Diseases for mental disorders [16]. A more recent study in Tunisia reported that the prevalence of homicide associated with mental illness was 14.7% between 2011 and 2018 [17]. Homicides associated with mental disorders were more prevalent in men than in women. Additionally, almost half of the offenders’ ages ranged between 18 and 35 in Wales and England, while more than half were younger than 30 years of age [18,19].

Psychotic disorders

A study conducted in England and Wales reported that, out of all homicide offenders, only 5% of them were diagnosed with schizophrenia [15]. In the aforementioned Russian study, 9.5% and 0.9% of mentally ill homicide offenders were diagnosed with schizophrenia and acute psychosis, respectively [16]. However, evidence linking higher rates of homicide and schizophrenia or bipolar disorder independently is lacking. Studies have shown a complex relationship between severe mental illness and committing homicide, as multiple risk factors play a role, including experiencing abuse in childhood, previous violent acts, substance abuse, homelessness, unemployment, and divorce, as well as the impact of the availability of mental healthcare services [20,21]. In another study, homicide risk factors in patients with schizophrenia and other psychoses were divided into three categories; before, during, and after admission. Risk factors before admission included poor self-care, unemployment, substance abuse, forced hospitalization, and hospitalization due to violence. However, the risk factors during admission included a history of severe mental illness for one year prior to hospitalization. Finally, nonadherence to medications and substance abuse were classified as after admission risk factors. Surprisingly, the presence of delusions or hallucinations was not a risk factor [22]. A German study compared the rate of homicide in patients with schizophrenia during two different periods (1955-1964 and 1992-1996). During the first period, the study reported that 8.20% of homicide offenders had schizophrenia, whereas 10% were reported between 1992 and 1996. Despite the differences in percentages, the odds ratios for both periods did not differ significantly. The positive reduction in rates of violence among patients with schizophrenia was proportionate to the increase in mental health services, but most importantly, to their quality [23]. Considering that schizophrenia is a chronic relapsing disorder, long-term follow-up is crucial, even in patients who appear well. This includes not only biological interventions but also the psychosocial aspects of management [24].

Mood disorders

In a retrospective study conducted in Iran, 20% of 600 hospitalized patients committed homicide. Of those, 20% were found to have bipolar disorders [19]. The manic phase of bipolar disorders was linked to a higher rate of violent offenses than the depressive phase (45.3% vs. 6.9%) in South Korea. In the same study, the risk of offending was estimated to be 23.7 times higher in the manic phase than in the depressive phase. However, this was not the case for homicide rates, as offenders in the manic phase were less likely to commit homicide than offenders in the depressive phase (61.1% vs. 92.3%) [25]. The rates of homicide related to unipolar depression were low [22]. Case linkage studies in Australia and Sweden reported that 2% of homicide offenders had depression [16,26,27]. Results from studies based on clinical examination of offenders were more diverse with regional differences: 9% in Singapore and the United States, 5% in Finland, 4% in England and Wales, 1.4% in Austria, and 1% in New Zealand, indicating higher depression rates [5,28-32]. The lowest reported rate of offenders with mood disorders was 0.8% in Russia [16].

Personality disorders

Studies have shown that almost 30% of homicide perpetrators were diagnosed with a personality disorder in Sweden, 23% in the United States, 15.3% in Russia, and 9% in England and Wales [15,16,26,28]. Nevertheless, in Ethiopia, a low-moderate income country, the results of a cross-sectional study that involved consenting prisoners convicted of homicide showed that out of 316 offenders, only 16% met the Diagnostic and Statistical Manual of Mental Disorders, fourth edition criteria for personality disorders. Of those, only 1.89% had an antisocial personality disorder, and the most prevalent disorder was borderline personality disorder (36%) [33]. In contrast, a Swedish study that assessed 1625 homicide offenders concluded that 90% of them were diagnosed with a psychiatric disorder, with more than half having substance abuse or cluster B personality disorders [26].

Substance abuse

Substance abuse, in particular, is widely associated with homicides, notably alcohol abuse [34]. Based on case linkage studies, 20% and 5% of homicide offenders were diagnosed with substance abuse in Sweden and Australia, respectively [26,27]. Studies based on psychiatric evaluations of homicide offenders reported rates of 47% in the United States, 12% in Finland, 1.4% in Austria, and 0.7% in New Zealand [5,28-30]. However, results reported by a larger-scale study in Russia, derived from file examinations of those who underwent judicial psychiatric examination, showed that 31.4% out of 46.7% of offenders were alcohol-dependent and 0.6% were opiate-dependent [16]. Moreover, according to statistics from the Journal of Studies on Alcohol and Drugs, rates of violence among men and women with untreated substance abuse were 72% and 50%, respectively [35]. Studies have suggested that intoxication lowers inhibition and predisposes mentally ill and healthy individuals to risky behavior [36-38]. In Saudi Arabia, a study conducted in Al-Taif, which examined the social and clinical differences among schizophrenic patients who committed homicide, revealed substance use and positive urine drug test results after crimes in 43.7% of homicide cases (56/128 cases) [39].

Organic disorders

Variations in the rates of organic mental disorders, brain damage, and epilepsy have been reported. For example, in Australia, 7% of homicide perpetrators suffered from pre-existing organic mental disorders, and some were found to have a history of traumatic brain injury; a higher rate was reported in Russia (15.5%) [15,26]. In contrast, a Swedish study reported no cases of organic mental disorders [25]. Additionally, intellectual disability among homicide perpetrators varied as follows: 7.7% in Russia, 2% in the United States and Finland, and less than 1% in Sweden and New Zealand [5,16,26,28,29].

Offenders’ motives and victims’ characteristics

Studies on the motives and intentions of homicides among mentally ill individuals are limited and have not been given enough attention compared to medically stable individuals. A study conducted in Sweden examined the motives of 48 offenders with schizophrenia who committed homicide between 1992 and 2000. The results showed that 26 offenders reported active psychosis while committing the crime, 13 were under alcohol or drug intoxication, and the remaining nine offenders’ motives were difficult to detect due to data limitations [40]. Another study conducted in Australia gathered all murder case convictions between 1997 and 2005. Motives for offenders with psychosis were divided into six categories: revenge, arguments, financial gain, sexual, sadistic, and filicide. Revenge was the top motive (54.8%), followed by arguments (38.1%), whereas sexual motives were insignificant in psychotic patients (0%) [41]. Another study analyzed all reports of homicides among women with personality disorders or psychosis between 1982 and 1992. Fighting with the victim was the most frequent motive (59%), followed by impulsive acts (30%), victim’s provocation (30%), long-standing violence from the victim (29%), extended suicide (11%), sexual motivation (11%), jealousy (10%), and self-defense (9%). Results from a Finnish study showed that victims are primarily individuals known by the offender; therefore, individuals with a close relationship with the offender have a higher risk of being the victims than strangers. Victims were divided into the following groups, from largest to smallest: intimate partners, former or present (54.4%), friends or acquaintances (24%), children (13.6%), parents and relatives (6.4%), and strangers (1.6%). The total number of adult victims was 108, and the number of child victims was 19 [42].

Methods of homicide

Generally, a vast number of methods are used in homicides and other criminal acts. The three most common means of homicide in the United States are firearms, piercing/cutting instruments, and suffocation [43]. Firearms were also documented as the most common method used for homicide in Ribeirao Preto, Brazil [44]. However, these methods are less commonly used in India, as blunt weapons are used more frequently. Burns and poisoning were the least commonly used methods. Furthermore, strangulation was often used in crimes where the victims were women [45]. Unlike the general population, the means of homicides used by individuals with mental illness were notably different. Sharp instruments (i.e., knives) were more commonly used than firearms in homicides. Firearms accounted for the least number of homicides in patients with severe mental illness [40]. These findings oppose the stigmatized public and media opinion about psychiatric disorders and their relation to gun violence, as it was proven that there is no relation between the increased risk of gun violence and suffering from mental illness. Access to firearms was an important factor related to gun violence, independent of the presence or absence of mental illness [46]. Following sharp instruments, different types of blunt objects, as well as strangulation, were used less frequently. Strangulation, suffocation, drowning, and burning have also been reported [47]. Several studies have been conducted to assess the correlation between different psychiatric disorders and the type of weapons used in homicides. Interestingly, psychotic disorders, particularly schizophrenia, are primarily associated with using sharp instruments [48,49]. The results of another study conducted in Saudi Arabia also support this finding: 57.8% of schizophrenic patients used sharp instruments, while 21.1% used firearms [38]. In addition, other disorders were associated with different methods. For example, mood disorders were related to strangulation, suffocation, and drowning. Alcohol-dependent individuals were more likely to kick and hit their victims, while organic disorders were linked to the use of blunt instruments [49].

## Conclusions

Studies investigating the link between major mental disorders and homicide have shown that schizophrenia was associated with the highest rate of homicide. In contrast, mood and anxiety disorders were associated with the lowest rate. Although the number of homicide offenders with mental disorders cannot be neglected, multiple confounders affect the relationship between homicide rates and mental illness, such as a history of an abusive childhood, previous violent acts, substance abuse, homelessness, unemployment, and divorce. Additionally, the stigma of mental illness seems to be the root of most, if not all, of these factors. Another possible factor that might have influenced the comparison of homicide rates in this review is the methodology of the studies. Although all the studies mentioned earlier focused on assessing the link between homicide and mental illness, each study used a different method. This factor should be considered when comparing homicide rates, as different study methods might alter the results. Interestingly, contrary to the common belief that gun violence is related to mental illness, studies have shown that none of the mental disorders were related to firearm use in homicides. Access to firearms was a fundamental factor in gun violence, regardless of the presence or absence of a mental disorder. Finally, studies on victim characteristics have shown that mentally ill offenders’ victims were individuals in close relationships with them, such as family members or friends, rather than strangers.

In summary, the number of homicides related to severe mental illness can be prevented by earlier and more effective treatment and follow-up, as well as the implementation of ways to reduce the stigma and discrimination toward mental illness in the public eye. Future research should explore the exact link between homicide and mental illness and implement intervention strategies to reduce both public and self-stigma. This will overcome the concept of associating mental illness with threat and danger.
